# Association of the *EPAS1* rs7557402 Polymorphism with Hemodynamically Significant Patent Ductus Arteriosus Closure Failure in Premature Newborns under Pharmacological Treatment with Ibuprofen

**DOI:** 10.3390/diagnostics13152558

**Published:** 2023-08-01

**Authors:** Diana G. Rogel-Ayala, José Esteban Muñoz-Medina, Valeria Dejanira Vicente-Juárez, Patricia Grether-González, Deneb Algedi Morales-Barquet, Alfonso de Jesús Martínez-García, María Olga Leticia Echaniz-Aviles, Rosalba Sevilla-Montoya, Alejandro Martínez-Juárez, Jazmin Artega-Vázquez, Javier Angeles-Martínez, Gilberto Vargas-Alarcón, Alberto Hidalgo-Bravo, Irma Eloisa Monroy-Muñoz

**Affiliations:** 1 Reproductive and Perinatal Health Research Department, National Institute of Perinatology, Mexico City 11000, Mexico; diana_gaby.rogel@hotmail.com (D.G.R.-A.);; 2 Quality of Supplies and Specialized Laboratories Coordination, Mexican Social Security Institute, Mexico City 37320, Mexico; 3 ABC Medical Center, Santa Fe, Mexico City 05348, Mexico; 4 Neonatal Intensive Care Unit, National Institute of Perinatology, Mexico City 11000, Mexico; deneb24@hotmail.com; 5 Fetal Maternal Medicine Department, National Institute of Perinatology, Mexico City 11000, Mexico; alfonsomg@gmail.com; 6 Newborn Intermediate Care Unit, National Institute of Perinatology, Mexico City 11000, Mexico; 7 Cellular Physiology Department, National Institute of Perinatology, Mexico City 11000, Mexico; 8 Department of Genetics, National Institute of Medical Science and Nutrition, Mexico City 14080, Mexico; 9 Specialized Laboratories Division, Mexican Social Security Institute, Mexico City 06700, Mexico; 10 Department of Molecular Biology, National Institute of Cardiology, Mexico City 14080, Mexico; 11 Genomics Medicine Department, National Institute of Rehabilitation, Mexico City 14610, Mexico

**Keywords:** *EPAS1* polymorphism, preterm infants, patent ductus arteriosus, pharmacological closure, ibuprofen

## Abstract

Patent ductus arteriosus (PDA) is frequent in preterm newborns, and its incidence is inversely associated with the degree of prematurity. The first choice of pharmacological treatment is ibuprofen. Several genes, including *EPAS1*, have been proposed as probable markers associated with a genetic predisposition for the development of PDA in preterm infants. *EPAS* 1 NG_016000.1:g.84131C>G or rs7557402 has been reported to be probably benign and associated with familial erythrocytosis by the Illumina Clinical Services Laboratory. Other variants of *EPAS1* have been previously reported to be benign for familial erythrocytosis because they decrease gene function and are positive for familial erythrocytosis because the overexpression of *EPAS1* is a key factor in uncontrolled erythrocyte proliferation. However, this could be inconvenient for ductal closure, since for this process to occur, cell proliferation, migration, and differentiation should take place, and a decrease in *EPAS1* gene activity would negatively affect these processes. Single-nucleotide polymorphisms (SNPs) in *EPAS1* and *TFAP2B* genes were searched with high-resolution melting and Sanger sequencing in blood samples of preterm infants with hemodynamically significant PDA treated with ibuprofen at the National Institute of Perinatology. The variant rs7557402, present in the *EPAS1* gene eighth intron, was associated with a decreased response to treatment (*p* = 0.007, OR = 3.53). The SNP rs7557402 was associated with an increased risk of pharmacological treatment failure. A probable mechanism involved could be the decreased activity of the product of the *EPAS1* gene.

## 1. Introduction

The ductus arteriosus is a central vascular shunt connecting the pulmonary artery to the aorta, allowing oxygenated blood from the placenta to bypass the uninflated fetal lungs and enter the systemic circulation. The rapid closure of the ductus after birth is essential for vascular transition to the mature divided pattern of arteriovenous circulation. Failed ductus arteriosus closure, termed patent ductus arteriosus (PDA), is frequent in preterm newborns, with up to 64% of infants born at 27 to 28 weeks exhibiting PDA. The incidence of PDA is inversely associated with the degree of prematurity [1,2].

The National Institute of Perinatology is a tertiary-level hospital, where 300 preterm newborns weighing <1500 g are born each year. An observational study performed in 2016 in 295 preterm infants between 27 and 28 weeks found PDA in 21.6% of patients [3]. The diagnostic gold standard is two-dimensional color Doppler echocardiography, which can determine the shape and diameter of the ductus arteriosus at the aortic and pulmonary edges and the degree of hemodynamic burden [4,5,6].

When there is hemodynamically significant PDA, the treatment scheme involves pharmacological treatment as the first choice. When the patient has a condition that contraindicates the use of drugs or if this treatment is not effective, surgical closure may be necessary. Untreated hemodynamically significant PDA can result in life-threatening conditions, such as congestive heart failure, pulmonary artery hypertension, and neonatal necrotizing enterocolitis [7].

Cyclooxygenase (COX) inhibitors are administered as a pharmacological treatment for hemodynamically significant PDA: the most commonly used inhibitors are indomethacin and ibuprofen, although the use of acetaminophen has recently been approved [8,9,10,11,12].

Up to 30% of pharmacological treatment failure has been observed in preterm infants. Both indomethacin and ibuprofen have shown similar efficacy [8,9,10,11,12]. In the National Institute of Perinatology, the most commonly used pharmacological treatment is ibuprofen. In the aforementioned study at the National Institute of Perinatology, 47.7% success was found with 10 mg/kg/d ibuprofen on the first day followed by 5 mg/kg/d ibuprofen on the second and third days, respectively, and 42.8% success was observed during the second cycle [3].

The ductus arteriosus (DA) derives from the left sixth aortic arch, which derives from neural crest cells. To successfully achieve ductal closure, cell growth and differentiation are essential, involving the induction of specific gene expression mediated by the interaction of transcription factors with response elements [13,14]. AP-2 transcription factors play a major role in the cellular differentiation induced by retinoic acid, particularly in neural crest cells. PDA probably results from the abnormal development of the neural crest [15].

Transcription factor AP-2 beta (*TFAP2B*) expression is enriched in the neural crest and may play an important role in regulating DA closure. This transcription factor has been reported to regulate the expression of *EPAS1* (endothelial PAS domain protein 1, also known as hypoxia-inducible factor 2 alpha), which is involved in oxygen sensing [2,16].

The expression of *EPAS1* is cell-type-restricted and predominantly occurs in endothelial cells, lung epithelial cells, and cardiac myocytes. *EPAS1* trans-activated target genes contain the hypoxia responsive element (HRE) [17,18].

A bioinformatic and statistical study led by Dagle in 2009 reported several genes as probable markers associated with the genetic predisposition to PDA in preterm infants. Of all possible markers analyzed, those that showed a significant association were the polymorphisms rs987237 of *TFAP2B* (*p* < 0.005) and rs1867785 of *EPAS1* (*p* < 0.005). These same polymorphisms were studied in relation to the failure of pharmacological treatment with indomethacin, also exhibiting significant association [13].

NG_016000.1:g.84131C>G or rs7557402 has been reported to be probably benign and associated with familial erythrocytosis by the Illumina Clinical Services Laboratory [19]. Other variants of *EPAS1* have been previously reported as benign for familial erythrocytosis because they decrease gene function, and they have been revealed to be positive for familial erythrocytosis because the overexpression of *EPAS1* is a key factor in uncontrolled erythrocyte proliferation [20]. However, this could be inconvenient for ductal closure, since for this process to occur, cell proliferation, migration, and differentiation should take place, and a decrease in *EPAS1* gene activity would negatively affect these processes [21,22,23].

In this study, we explored the association between genetic variants of *TFAP2B* and *EPAS1* and PDA ibuprofen treatment failure.

## 2. Materials and Methods

### 2.1. Study Population

A cross-sectional exploratory study including 47 newborns with a diagnosis of PDA treated with ibuprofen was carried out at the National Institute of Perinatology in Mexico City. On behalf of the children enrolled in our study, we obtained written informed consent from their legal guardians. The study complied with the Declaration of Helsinki and was approved by the Institutional Ethics Committee and registered to the National Institute of Perinatology, project number 212250-3140-11105-01-16.

Convenience sampling of consecutive cases was performed. All premature newborns (gestational age between 25 and 36.6 gestation weeks determined by Ballard) with hemodynamically significant PDA diagnosis and ibuprofen treatment born at INPer during the period from 1 January 2017 to 31 December 2019 were included. Oral ibuprofen treatment was used as follows: the first cycle of 10 mg/kg/d on the first day, followed by 5 mg/kg/d on the second and third days, respectively. In the case that no clinical and echocardiographic response was obtained (Supplementary Materials), a second oral ibuprofen cycle was administered (20 mg/kg/d), followed by a second and third dose at 10 mg/kg/dose, with administration intervals of 24 h between the doses [3]. The sample population was separated into two groups. The case group (*n* = 19) included patients who presented ibuprofen treatment failure, and hemodynamically significant PDA was closed via surgery. The control group (*n* = 28) included patients with successful pharmacological treatment with ibuprofen.

In addition to the premature newborn samples, we included a family of nine members with a background of PDA. Blood samples from members of this family were used as controls for the techniques employed in this study.

### 2.2. Genetic Variant Analysis

A sample of peripheral blood was taken with a BD Microtainer^®^ (Franklin Lakes, NJ, USA) blood collection system. DNA was isolated from leukocytes using the Promega Wizard^®^ Genomic DNA Purification Kit (Madison, WI, USA). The DNA samples were quantified with a Thermo Scientific NanoDrop™ 2000 instrument (Waltham, MA, USA) and aliquoted and stored at −20 °C until later use.

The high-resolution melting (HRM) method was used to detect genetic variants of the *TFAP2B* and *EPAS1* genes. The HRM primers were designed using the Primer Select program (DNASTAR lasergene, Maddison, WI, USA), which also ensures that the primer sequences do not form secondary structures during PCR that could increase the complexity of melting profile interpretation. The primer specificity was tested using the Primer Blast platform (NCBI) [24]. Primers were designed to amplify complete exonic sequences and small flanking intronic sequences. Reactions were performed with a total volume of 20 µL (5 µL of Milli-Q water, 1.5 µL of each primer at 20 pmol/µL, 10 µL of Bio-Rad Precision Melt Supermix (Hercules, CA, USA), and 2 µL of DNA at 150 ng/µL). The amplification parameters were 95 °C for 4 min, 30 cycles of 94 °C for 30 s, annealing temperature for 30 s, and 72 °C for 30 s, followed by a final extension step of 72 °C for 5 min. For the melting curve analysis, the parameters were 95 °C for 30 s and 75 °C for 30 s. Data were collected over a temperature range of 75–95 °C in 0.1 °C increments every 10 s using the CFX-96 Touch Real Time PCR System of Bio-Rad (Hercules, CA, USA). Once each HRM reaction was standardized, all samples were tested in triplicate. Melting curve analysis was performed with Bio-Rad Precision Melt Analysis Software v.1.3. (Hercules, CA, USA). All samples with melting curves different from the average underwent capillary sequencing to determine the alteration in the sequence that yielded the difference in the melting curve (see Supplementary Materials). Moreover, three random samples with an average melting curve were capillary sequenced to ensure that the observed curve corresponded to the consensus sequence obtained from the RefSeq database (NCBI) [25].

### 2.3. DNA Sequencing

After purification with Thermo Fisher Scientific PCR ExoSAP-IT^TM^ (Waltham, MA, USA), HRM products were sequenced using Applied Biosystems Big Dye Terminator v1.1 and v3.1 kits (Applied Biosystems, Foster City, CA, USA) and an ABI PRISM 3130 DNA Analyzer (Applied Biosystems, Foster City, CA, USA). The obtained sequences were analyzed with BioEdit v.7.2 software (Ibis Biosciences, Carlsbad, CA, USA) and the NCBI Nucleotide Blast platform (Blastn) [26]. Nucleotide sequences were translated using the translate tool in the ExPASy Bioinformatics Resource Portal [27]. Whenever a sequence variant was found, the sample was sequenced again from the opposite direction to confirm the nucleotide change.

### 2.4. Bioinformatic Analysis

All variants found were searched in the NCBI databases dbSNP [28] and ClinVar [29] to gather information from previous reports. They were also analyzed with Mutalyzer v.3. software [30] to predict whether they could produce a change in the protein sequence.

The variants that were predicted to alter the protein sequence were analyzed with the PolyPhen v2.0.23 bioinformatic tool [31], which can classify those changes as probably damaging, possibly damaging, or benign. Predictions are based on multiple alignments of proteins that are closely related in function and amino acid sequence to the tested protein.

This tool also considers all available data in the protein databases UniProt [32] and RCSB [33] PDB, such as tridimensional structures and specific protein domain information.

Synonymous variants were analyzed with Human Splicing Finder v.3.0 (HSF) [34], which integrates internal and external detection matrices of splicing sites, branch points, splicing regulatory sequences, etc., to detect site break canonical splicing acceptors and donors, create alternative donor and acceptor sites, analyze branch point breaking, create silencers, and remove enhancers.

### 2.5. Statistical Analysis

Allele and genotype frequencies of the studied polymorphisms were obtained via direct counting. The Hardy–Weinberg equilibrium (HWE) was calculated using the ꭓ^2^ test. The significance of the difference between groups was determined using Mantel–Haenzel chi-square analysis. All calculations were performed using SPSS version 18.0 (SPSS, Chicago, IL, USA). Means + SDs and the frequencies of baseline characteristics were calculated. Student’s *t*-test was performed to compare differences between continuous variables, and the categorical data were analyzed using ꭓ^2^ and Fisher exact tests. Logistic regression analysis was used to test for the association of polymorphisms with clinical variables under dominant, recessive, codominant, and additive inheritance models in the independent analysis. The most appropriate inheritance model was selected based on Akaike information criteria and was adjusted for gestational age and sex. The statistical power to detect associations with clinical variables was >0.80, as estimated with QUANTO v.1.2.4 software [35]. Pairwise linkage disequilibrium (LD, D’) estimations between polymorphisms and haplotype reconstruction were performed with Haploview version 4.1 [36] (Broad Institute of Massachusetts Institute of Technology and Harvard University, Cambridge, MA, USA).

## 3. Results

### 3.1. Population Sample Characteristics

A population sample of 47 patients with a diagnosis of hemodynamically significant PDA participated in the study, and they were divided into two groups: 19 cases (11 males and 8 females) and 28 controls (19 males and 9 females) (Table 1).

Gestational age and birth weight are important risk factors for developing PDA; for this reason, it was necessary to ensure that they were comparable between groups (Table 1). No statistically significant differences were found regarding gestational age (*p* = 0.112) (Table 1). A significant difference was found with respect to birth weight (*p* < 0.0001). Therefore, further analysis was adjusted for this variable to avoid spurious results.

We also found statistically significant differences in sepsis development (*p* < 0.0001), with or without taking the time of development into account, but it was more significant in cases of late development, as observed in Table 1.

### 3.2. Genetic Variant Detection

We found 63 genetic variants in the population sample: 24 only in the case group, 26 only in the control group, and 13 in both groups (Table 2).

Of the 24 variants found in the case group, 13 were synonymous, 6 were missense, and 5 were INDEL. Only 11 variants resulted in changes in the sequence of amino acid residues in the protein.

In the control group, we found 19 synonymous variants, 7 missense variants, and no INDEL variants (26 altogether, as mentioned before). In this group, only 7 variants affected the protein.

The frequency of genetic variants that modify the sequence of amino acids was higher in the case group, especially considering that this group is smaller. Moreover, this group contained all INDEL variants found. INDEL variants generally cause a shift in the open reading frame, completely changing the amino acid sequence, and can result in premature stop codons.

The statistical analysis of the variants shared by both groups showed no association of *TFAP2B* variants with treatment response (Table 2).

Only the variant NG_016000.1:g.84131C>G, present in the *EPAS1* gene, eighth intron, previously reported as rs7557402, was associated with a risk factor for failure to respond to treatment (*p* value of 0.007, OR of 3.53, and 95% confidence level). It was found in both groups of the study and in the samples of the PDA family used for the standardized techniques employed in this study.

Because we found the three genotypes present in our study population, it was possible to test haplotype association with treatment response, finding that the recessive model (homozygosity) was strongly associated with treatment failure (*p* value of 0.017 and 95% confidence level), unlike the dominant and codominant models, which were not statistically associated (Table 3).

According to the Mutalyzer analysis [30], this variant produces no change in amino acid sequence; therefore, it could not be analyzed using the PolyPhen2 bioinformatic tool [31].

**Figure 1:**
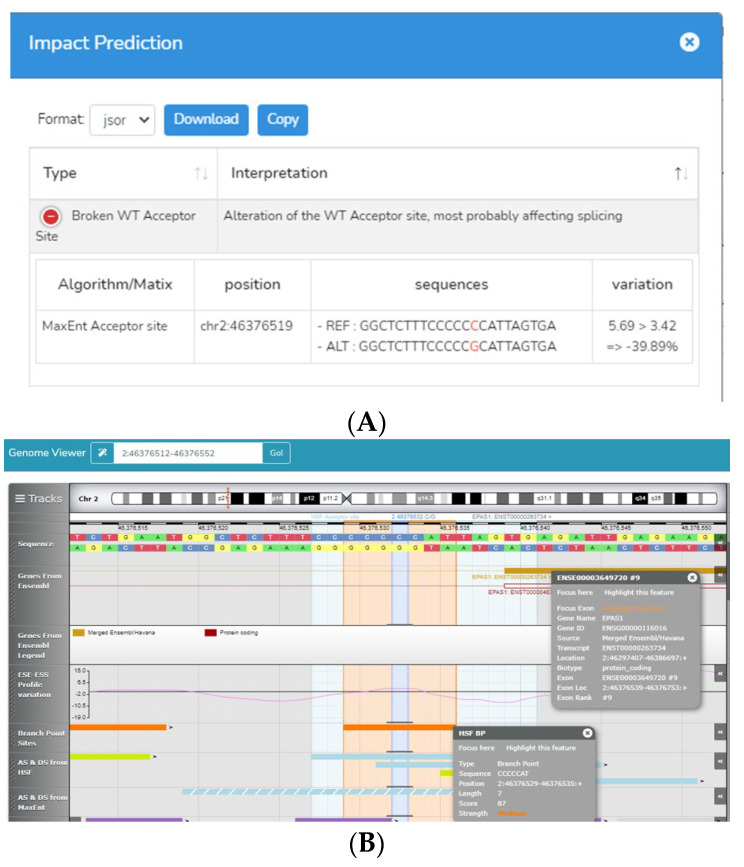
Prediction of the NG_016000.1:g.84131C>G variant effect made with HSF3 v. 3.0. software. (**A**) Impact prediction showed that this variant alters a wild-type acceptor site by disrupting it. (**B**) The genome viewer showed that the site where the variant is located is an acceptor site with a medium force, whose disruption most likely affects the splicing of the *EPAS1* gene 8th intron.

Instead, it was analyzed with Human Splicing Finder 3.0 (HSF) [34]. HSF analysis showed that this nucleotide change generates the disruption of the wild-type acceptor splice site located in the intron 8 acceptor splice site (Figure 1).

This variant has been reported as probably benign for familial erythrocytosis by the Illumina Clinical Services Laboratory. The classification does not have a clinical basis, but a criteria classification basis, according to ACGM (American College of Medical Genetics and Genomics) guidance [37].

**Figure 2:**
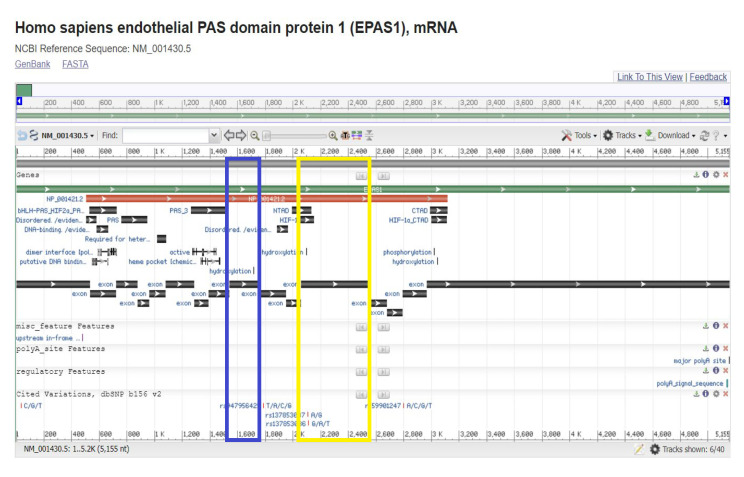
Analysis of the codifying sequence of *EPAS1*. The codifying sequence NM_001430.5 showed the presence of two hydroxylation sites, the first one located in exon 9, highlighted with the blue rectangle, and the second one highlighted with the yellow rectangle. Both sites encode a 4-hydroxyproline.

The analysis of the codifying sequence of *EPAS1* showed that exon 9 codes for a hydroxylation site and that it is necessary for the covalent post-translational processing of the transcription factor (Figure 2).

## 4. Discussion

PDA has a high frequency in the Mexican population, especially in preterm infants [3]. At birth, hemodynamically significant PDA is treated with COX inhibitors, and oral ibuprofen is the treatment of choice at the National Institute of Perinatology, but the use of these types of drugs generates adverse effects that can further complicate patient health. Therefore, the search for biological markers for predicting the efficacy of pharmacological treatment in preterm newborns could avoid unnecessary exposure to the adverse effects of drugs. *TFAP2B* is the most studied gene in the development of PDA, since it was described as being part of Char syndrome, the phenotype of which may include PCA [38,39]. Nevertheless, the use of massive sequencing and bioinformatic tools has made it possible to identify new target genes for this disease, such as *EPAS1*. This gene encodes a transcription factor induced when oxygen levels fall. It is part of the molecular pathway necessary to close the ductus arteriosus and is regulated by oxygen levels [40]. A higher number of variants in both genes in the case group and the presence of variants such as rs7557402 indicates a weak but measurable association with a lack of response to pharmacological treatment, highlighting the need for the further and deeper study of this population.

The rs7557402 variant has been reported as probably benign according to the American College of Medical Genetics and Genomics (ACGM) criteria. This variant has been associated with familial erythrocytosis by the Illumina Clinical Services Laboratory [21]. Some other variants in *EPAS1* have been previously reported as benign and are also associated with familial erythrocytosis because they decrease gene product function. These variants have a positive effect on familial erythrocytosis since the overexpression of *EPAS1* is a key factor for uncontrolled erythrocyte proliferation [20].

It is possible that the variant described as “benign” for familial erythrocytosis may be associated with a decrease in EPAS1 activity. This effect is inconvenient for ductal closure, which requires cell proliferation, migration, and differentiation. A decrease in *EPAS1* gene activity would negatively affect all of these processes [22,23]. The activity of EPAS1 could be diminished since the analysis with HSF3 v. 3.0. software showed that the C>G substitution in intron 8 leads to the breakdown of an acceptor site. The ultimate effect is faulty splicing of the mRNA, and therefore a defective protein.

Inside the *EPAS1* exon 9 sequence, there is a hydroxylation site. *EPAS1* (or *HIF-2α)* belongs to the family of hypoxia-inducible factors (HIFs). These factors are formed by two subunits, one with nuclear localization HIF-β and the other with cytoplasmic localization HIF-α. HIF-2α possesses 48% amino acid identity with HIF-1α; it is regulated by prolyl hydroxylation, dimerizes with HIF-2β, and binds to the same target DNA sequence (5′-RCGTG-3′) as the HIF-1α:HIF-1β heterodimer. The sets of genes regulated by HIF-1 and HIF-2 overlap, but some are specific and depend on cell type [41]. At physiological oxygen levels (normoxia), HIF-prolyl hydroxylases (PHDs) hydroxylate proline residues on HIF-α subunits, leading to their destabilization by promoting ubiquitination via the von Hippel Lindau (VHL) ubiquitin ligase and subsequent proteasomal degradation [42]. Functional specificity for transactivation via HIF-1α and HIF-2α appears to reside in amino acid residues 415 to 659 and 418 to 619, respectively [41]. HIF-α transactivation is also repressed in an O_2_-dependent manner due to asparaginyl hydroxylation via the factor-inhibiting HIF (FIH). In hypoxia, the O_2_-dependent hydroxylation of HIF-α subunits via PHDs and FIH is reduced, resulting in HIF-α accumulation, dimerization with HIF-β, and migration into the nucleus to induce an adaptive transcriptional response [42]. In this case, the covalent modification determines where the protein should be located according to the cellular conditions. HIF-2α hydroxylation sites are located inside exons 9 and 12 (Figure 2). However, how these hydroxylation sites are regulated through space and time is not very clear. One possibility is that the rs7557402 variant disrupts the hydroxylation site encoded in exon 9, leading to the increased translocation of HIF-2α into the nucleus and triggering the expression of genes related to the hypoxia response, which will result in the opposite response as expected. Another possible explanation could be related to a reduced rate of HIF-2α degradation secondary to deficient ubiquitination and degradation by the proteasome. The lack of renewal of HIF-2α could compromise its function. Under normal conditions, HIF-α is continuously synthesized and degraded [42]. In addition to PHD-conferring alterations in protein stability, there is now evidence that hydroxylation can affect protein activity and protein/protein interactions with respect to alternative substrates; therefore, if the hydroxylation sites are lost, HIF-2α activity could be affected [41].

The variant rs7557402 has a CADD score for the G allele of 7.555; this score indicates that the variant is likely benign. In addition, the GERP score is 1.33, which means that the variant is highly conserved among species. We performed an alignment including ten species of mammals, and all of them but the *capra hircus* showed conservation of the C allele [43]. Furthermore, the analysis with RegulomeDB v.2 data showed that the rs7557402 variant generates more compacted chromatin, resulting in a weaker transcription of the *EPAS1* gene in the fetal heart [44].

It is also important to highlight the presence of rs7557402 in the samples of the family used for the standardization of the techniques employed in this study. These samples were not part of the study because we do not have enough information about the treatments employed before closure via catheterization for each case. Nevertheless, we could infer that they ultimately required invasive treatment for the closure of the PDA, as in our case group. Moreover, the presence of this variant in a family case corroborates the effects of genetic factors other than the *TFAP2B* gene on PDA development and, in this particular case, on the lack of response to pharmacological treatment and the need to close PDA using invasive treatment, such as surgery or catheterization.

These results should be interpreted with caution because of the limited sample size. This study must be replicated in a larger population to confirm or reject the association.

## 5. Conclusions

The rs7557402 SNP is associated with the failure of pharmacological treatment for PDA closure, specifically with ibuprofen in our study population. A possible explanation could be the decrease in the activity of the *EPAS1* gene associated with the variant. Further studies with a larger sample size are needed to strengthen this association and decide whether this polymorphism could be used as a prognostic biomarker for pharmacological response.

Moreover, our findings suggest the need to study other genetic factors involved in PDA development in addition to *TFAP2B* in preterm newborns.

## Figures and Tables

**Table 1:** Demographic characteristics of the population.

		Control	Case	*p*
**Mother**				
Hypertension *n* (%)		9 (32.1)	7 (36.8)	0.763
Preeclampsia *n* (%)		9 (32.1)	5 (26.3)	0.753
T2D *n* (%)		4 (14.3)	0	0.137
Prenatal infections *n* (%)		12(42.9)	9 (47.4)	0.775
Prenatal treatment	Indomethacin *n* (%)	0	1 (5.3)	0.404
	Steroid *n* (%)	20 (76.9)	13 (68.4)	0.734
**Newborn**				
Sex F/M *n*		9/19	8/11	0.546
Gestational age		30 ± 2.3	28.8 ± 2.8	0.112
Birth weight (g)		1229 ± 349	928 ± 154	**<0.0001**
Ductal diameter (mm)		2.9 ± 0.9	2.9 ± 0.8	0.765
Sepsis *n* (%)		16 (57.1)	1 (5.3)	**<0.0001**
	Early *n* (%)	7 (25)	0	**0.032**
	Late *n* (%)	10 (35.7)	0	**0.003**

Data are expressed as means ± SD or frequencies. The *p* values were calculated by using Student’s *t*-test and chi-square/Fisher exact test where appropriate to compare among groups. F: female, M: male. T2D: Type 2 Diabetes. Statistically significant values are highlighted in bold.

**Table 2:** Comparison of genotype and allele frequencies of variants between the case and control groups.

	Genotype Frequency (%)			*TFAP2B*	Allelic Frequency (%)					
**9757AT**	**A/A**	**A/T**	**T/T**	*p* *	**A**	**T**	*p* **	**ORc**	**(IC 95%)**	***p* HW**
Control (*n* = 28)	23 (82)	5 (18)	0 (0)		51 (91)	5 (9)				1
Case (*n* = 19)	19 (100)	0 (0)	0 (0)	**0.018**	38 (100)	0 (0)	**0.0032**	-	-	1
**29461AT**	**A/A**	**A/T**	**T/T**		**A**	**T**				
Control (*n* = 28)	24 (85.7)	4 (14.3)	0 (0)		52 (93)	4 (7)				1
Case (*n* = 19)	17 (89.5)	2 (10.5)	0 (0)	0.70	36 (95)	2 (5)	0.767	0.699	0.21–2.28	1
				***EPAS1***						
**rs7557402**	**C/C**	**C/G**	**G/G**		**C**	**G**				
Control (*n* = 28)	24 (85.7)	4 (14.3)	0 (0)		52 (93)	4 (7)				1
Case (*n* = 19)	14 (73.7)	2 (10.5)	3 (15.8)	0.05	30 (79)	8 (21)	**0.007**	**3.53**	**1.42–8.74**	0.0096
**54455C>T**	**C/C**	**C/T**	**T/T**		**C**	**T**				
Control (*n* = 28)	24 (85.7)	4 (14.3)	0 (0)		52 (93)	4 (7)				1
Case (*n* = 19)	17 (89.5)	1 (5.3)	1 (5.3)	0.25	35 (92)	3 (8)	1	1.15	0.402–3.31	0.08
**54522G>A**	**G/G**	**G/A**	**A/A**		**G**	**A**				
Control (*n* = 28)	24 (85.7)	1 (3.6)	3 (10.7)		49 (88)	7 (12)				0.0007
Case (*n* = 19)	16 (84.2)	0 (0)	3 (15.8)	0.53	32 (84)	6 (16)	0.541	1.39	0.62–3.12	0.0003
**rs769385664**	**G/G**	**G/A**	**A/A**		**G**	**A**				
Control (*n* = 28)	26 (92.6)	1 (3.6)	1 (3.6)		53 (95)	3 (5)				0.055
Case (*n* = 19)	16 (84.2)	3 (15.8)	0 (0)	0.21	35 (92)	3 (8)	0.567	1.65	0.521–5.23	1
**54699C>A**	**C/C**	**C/A**	**A/A**		**C**	**A**				
Control (*n* = 28)	21 (75)	5 (17.9)	2 (7.1)		47 (84)	9 (16)				0.11
Case (*n* = 19)	13 (68.4)	4 (21.1)	2 (10.5)	0.87	30 (79)	8 (21)	0.466	1.39	0.679–2.86	0.14
**54700A>C**	**A/A**	**A/C**	**C/C**		**A**	**C**				
Control (*n* = 28)	22 (78.6)	5 (17.9)	1 (3.6)		49 (88)	7 (12)				0.35
Case (*n* = 19)	14 (73.7)	4 (21.1)	1 (5.3)	0.92	32 (84)	6 (16)	0.541	1.39	0.623–3.127	0.37
**54701G>C**	**G/G**	**G/C**	**C/C**		**G**	**C**				
Control (*n* = 28)	19 (67.9)	8 (28.6)	1 (3.6)		46 (82)	10 (18)				1
Case (*n* = 19)	14 (73.7)	5 (26.3)	0 (0)	0.57	33 (87)	5 (13)	0.434	0.68	0.313–1.476	1
**rs1286681984**	**C/C**	**C/A**	**A/A**		**C**	**A**				
Control (*n* = 28)	24 (85.7)	4 (14.3)	0 (0)		52 (93)	4 (7)				1
Case (*n* = 19)	16 (84.2)	2 (10.5)	1 (5.3)	0.38	34 (89)	4 (11)	0.459	1.64	0.61–4.42	0.16
**54703C>G**	**C/C**	**C/G**	**G/G**		**C**	**G**				
Control (*n* = 28)	24 (85.7)	4 (14.3)	0 (0)		52 (93)	4 (7)				1
Case (*n* = 19)	17 (89.5)	2 (10.5)	0 (0)	0.7	36 (95)	2 (5)	0.767	0.699	0.21–2.28	1
**54707C>G**	**C/C**	**C/G**	**G/G**		**C**	**G**				
Control (*n* = 28)	25 (89.3)	3 (10.7)	0 (0)		53 (95)	3 (5)				1
Case (*n* = 19)	17 (89.5)	2 (10.5)	0 (0)	0.98	36 (95)	2 (5)	1	1	0.28–3.56	1
**rs762559920**	**G/G**	**G/C**	**C/C**		**G**	**C**				
Control (*n* = 28)	27 (96.4)	1 (3.6)	0 (0)		55 (98)	1 (2)				1
Case (*n* = 19)	16 (84.2)	3 (15.8)	0 (0)	0.14	35 (92)	3 (8)	0.1	4.26	0.88–20.59	1

Data are expressed as frequencies. The *p* values were calculated using * ꭓ^2^ or ** Fisher’s exact test. Statistically significant values are highlighted in bold.

**Table 3:** rs7557402 association analysis: case-control.

Model	Genotype	Case	Control	OR (95% CI)	*p* Value
**Codominant**	C/C	14 (73.7%)	24 (85.7%)		0.056
	C/G	2 (10.5%)	4 (14.3%)	1.17 (0.19–7.21)	
	G/G	3 (15.8%)	0 (0%)	0.00 (0.00-NA)	
**Dominant**	C/C	14 (73.7%)	24 (85.7%)		0.31
	C/G-G/G	5 (26.3%)	4 (14.3%)	0.47 (0.11–2.03)	
**Recessive**	C/C-C/G	16 (84.2%)	28 (100%)		**0.017**
	G/G	3 (15.8%)	0 (0%)	0.00 (0.00-NA)	
**Overdominant**	C/C-G/G	17 (89.5%)	24 (85.7%)	1	0.7
	C/G	2 (10.5%)	4 (14.3%)	1.42 (0.23–8.64)	
**Additive**	-	-	-	0.41 (0.13–1.26)	0.098

Statistical significant values are highlighted in bold.

## Data Availability

All data generated or analyzed during this study are included in this published article.

## Supplementary Materials

The following supporting information can be downloaded at: https://www.mdpi.com/article/10.3390/diagnostics13152558/s1. Supplementary Figure S1: Transthoracic echocardiogram with bidimensional and color Doppler modalities that shows a short-axis view of the heart with a hemodynamically significant patent ductus arteriosus. Supplementary Figure S2: Transthoracic echocardiogram with bidimensional and color Doppler modalities that shows a sagittal view of a hemodynamically significant patent ductus arteriosus. Supplementary Figure S3: Transthoracic echocardiogram with bidimensional and color Doppler modalities that shows a short-axis view of the heart of a patient that underwent pharmacologic treatment for the closure of the ductus arteriosus. The image shows laminar flow in the pulmonary artery without any residual shunt.

## Sample Availability

No samples are available from the authors.

## Abbreviations

The following abbreviations are used in this manuscript:

PDA	Patent ductus arteriosus
EPAS1	Endothelial PAS domain protein 1
SNP	Single-nucleotide polymorphism
OR	Odds ratio
HRM	High-resolution melting
HSF	Human splicing finder
ACGM	American College of Medical Genetics and Genomics
COX	Cyclooxygenase
TFAP2B	Transcription factor AP-2 beta
HIF	Hypoxia-inducible factor
PHD	Prolyl hydroxylase
