# Supplementary material for: Association of the EPAS1 rs7557402 Polymorphism with Hemodynamically Significant Patent Ductus Arteriosus Closure Failure in Premature Newborns under Pharmacological Treatment with Ibuprofen
Source: Diagnostics (Basel). 2023 Aug 1;13(15):2558. doi: 10.3390/diagnostics13152558 (PMC10417126; doi:10.3390/diagnostics13152558)
Supplement: Supplementary file 1 [file diagnostics-13-02558-s001.zip › diagnostics-2509576-supplementary.pdf]

Supplementary Materials

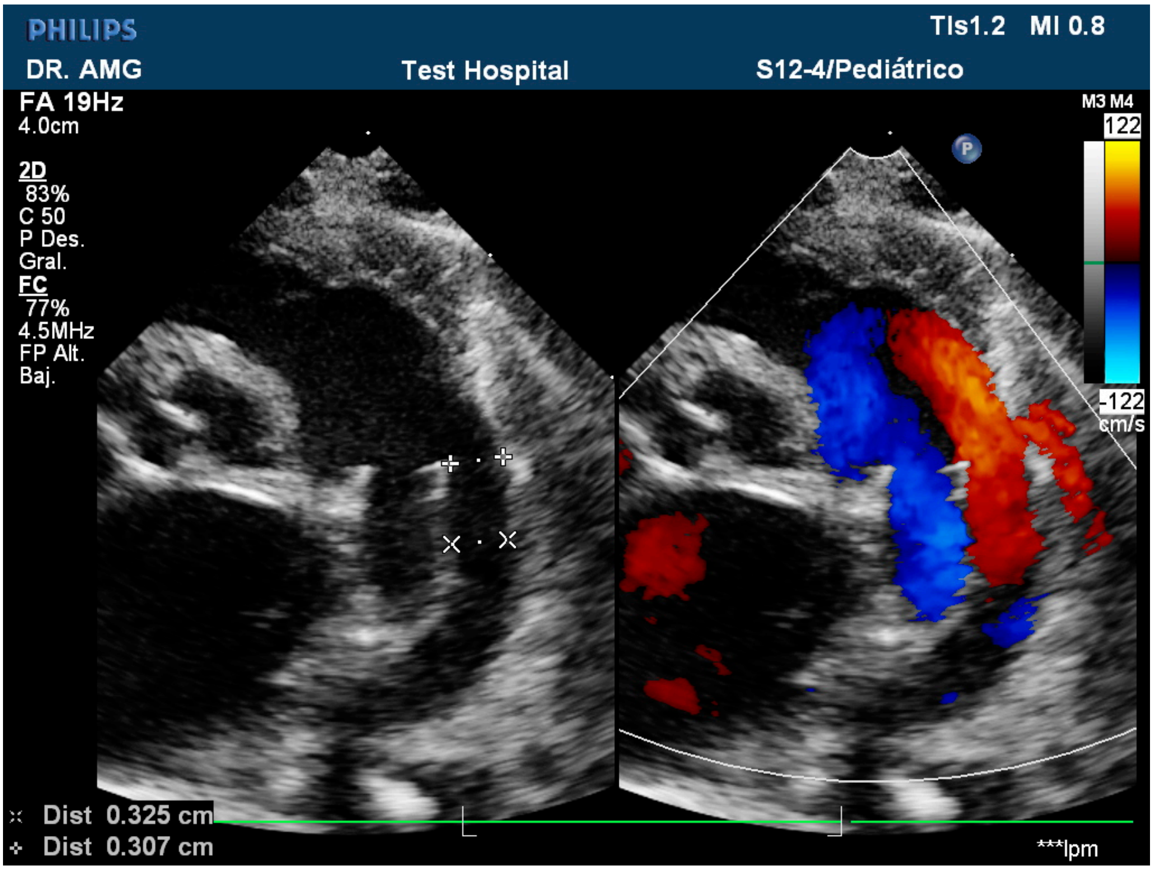

Figure S1. Transthoracic echocardiogram with bidimensional and color Doppler modalities that shows a short axis view of the heart with a hemodynamically significant patent ductus arteriosus.

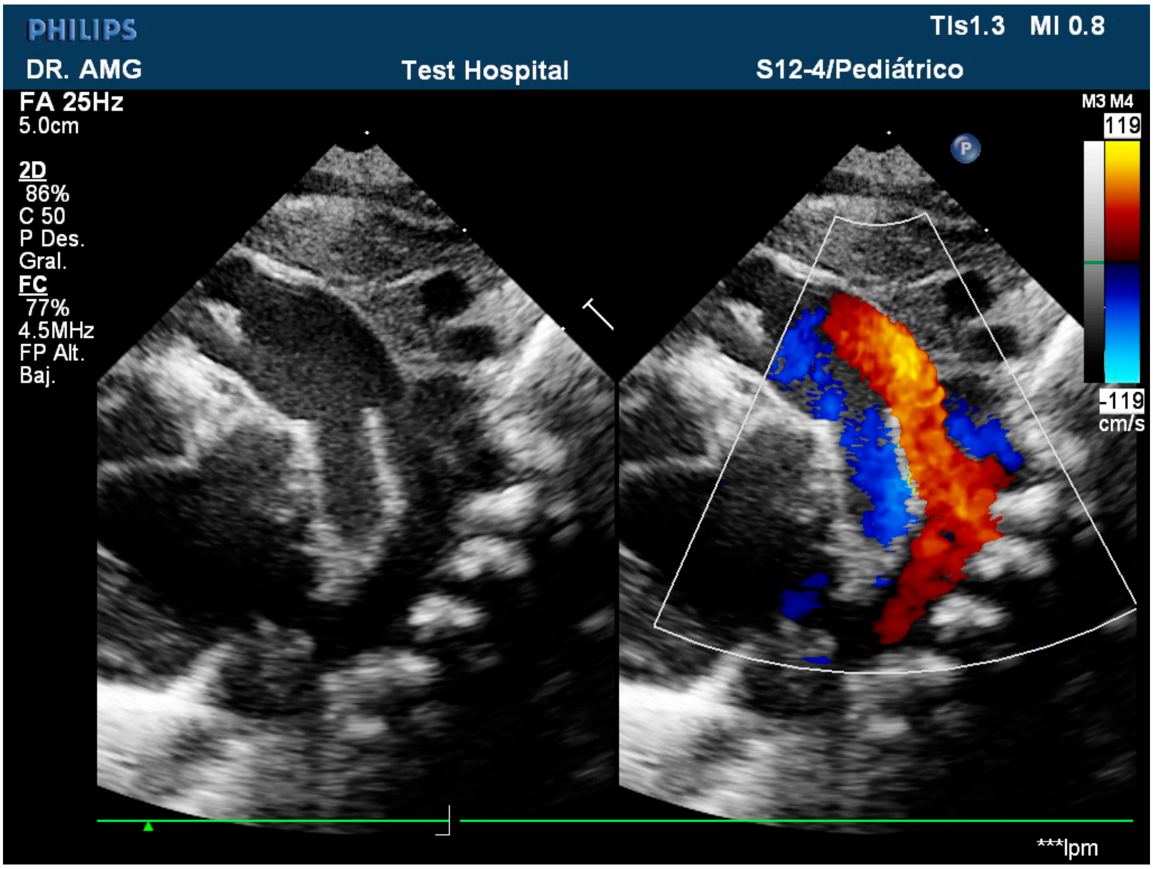

Figure S2. Transthoracic echocardiogram with bidimensional and color Doppler modalities that shows a sagittal view of a hemodynamically significant patent ductus arteriosus.

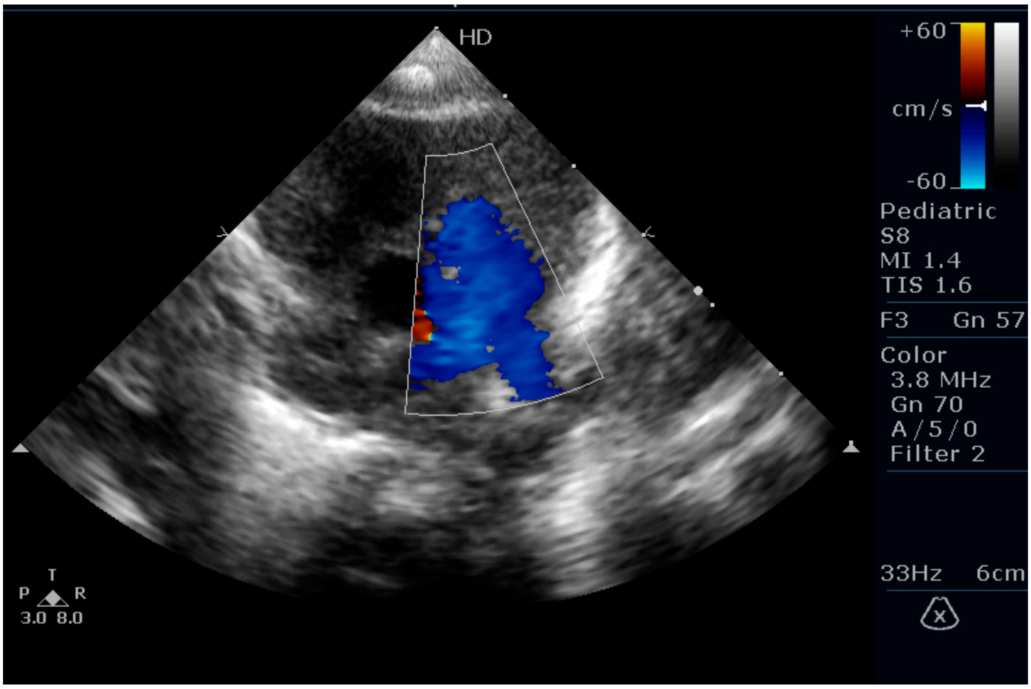

Figure S3. Transthoracic echocardiogram with bidimensional and color Doppler modalities that shows a short axis view of the heart of patient that underwent pharmacologic treatment for closure of the ductus arteriosus. The image shows laminar flow in the pulmonary artery without any residual shunts.
